# Acupuncture Use among American Adults: What Acupuncture Practitioners Can Learn from National Health Interview Survey 2007?

**DOI:** 10.1155/2012/710750

**Published:** 2012-02-22

**Authors:** Yan Zhang, Lixing Lao, Haiyan Chen, Rodrigo Ceballos

**Affiliations:** ^1^Division of Health Services Research, Department of Family and Community Medicine, Laura W. Bush Institute for Women's Health, Texas Tech University Health Sciences Center, 3601 4th Street, STOP 8143, Lubbock, TX 79430, USA; ^2^Family and Community Medicine, TCM Research Program, Center for Integrative Medicine, University of Maryland School of Medicine, East Hall, 520 W. Lombard Street, Baltimore, MD 21201, USA; ^3^Division of Health Services Research, Department of Health Promotion and Policy, University of Maryland School of Dentistry, and Division of Preventive Medicine, Department of Epidemiology and Public Health, University of Maryland School of Medicine, 650 West Baltimore Street, Room 2213, Baltimore, MD 21201, USA; ^4^Department of Family and Community Medicine, Paul L. Foster School of Medicine, Texas Tech University Health Sciences Center, 9849 Kenworthy Street, El Paso, TX 79924, USA

## Abstract

This paper examined the National Health Interview Survey (NHIS) 2007 and explored acupuncture users sociodemographics characteristics, reasons and the nature of acupuncture use, and the relationship of such use with conventional medical care. All individuals who completed adults core interviews (*N* = 23,393) were included. Three subsets of samples (nonuser, former user, and recent user) were used in the analysis performed in Stata. Our findings revealed that ever acupuncture user (including former and recent user) increased from 4.2% to 6.3% of the population, representing 8.19 million and 14.01 million users in 2002 and 2007, respectively. We expected this trend to continue. People not only used acupuncture as a complementary and alternative approach to conventional treatment for a specific health condition, but also used it as a preventive means to promote general health. Effectiveness and safety appeared not to be the main predictors of acupuncture use; rather, awareness, cost, and insurance coverage played a bigger role in decision making.

## 1. Introduction

The use of complementary and alternative medicine (CAM) in the American adult population increased substantially during the 1990s [1] and has remained at a relatively stable rate (36–38%) over the past 10 years [2–4]. In particular, the use of practitioner-based CAM modalities such as chiropractic care, massage, and acupuncture have increased significantly in the USA from 2002 to 2007 [5]. Acupuncture has attracted public attention as well as researchers' interest since it was introduced to the USA in1970s [6]. The National Health Interview Survey (NHIS) 2002 revealed that 1.1% (approximately two million) of the American adults had used acupuncture within the past 12 months [2, 7]. By 2007, this number had expanded to three million, a 50% increase in five years [3]. There were 79.2 visits to acupuncturists per 1000 adults in 2007, a nearly triple increase from the 27.2 visit reported in 1997 [8]. The acupuncture workforce has also been growing. As of 2003, there were 20,750 licensed acupuncturist in the USA [9], and based on the most recent available data, the number was estimated to be 28,000 licensed in 2009 and still keeps increasing [10]. However, when compared to other western countries, Americans are reported as having a lower prevalence of acupuncture use than their counterparts in Australia, UK, Norway, and Denmark [11–13]. Reasons for Americans using or not using acupuncture are worth exploring. A previous study reported that predisposing factors (e.g., ethnicity and education), enabling factors (e.g., region of residence), and medical need and personal health practices are associated with women's recent use of acupuncture services [14]. NHIS 2007 added new questions particularly pertaining to the reasons of “never used acupuncture” or “not used it the past 12 months” as well as patient's acupuncture use combined with other treatments. In this paper, we present the findings of our analysis of NHIS 2007 sample on acupuncture users sociodemographic characteristics, reason and nature of acupuncture use, and its relationship of such use with conventional Western medical care. We hypothesized that acupuncture users characteristics in 2007 would have some differences than that of acupuncture users in 2002. More importantly, we expected that the findings of this study would help acupuncture practitioners to learn more about the reasons for their patients' visit and the nature of acupuncture use so to provide better services accordingly.

## 2. Materials and Methods

### 2.1. Data Sources

This study is a secondary analysis of extant survey data of NHIS 2007. The current analysis utilized the datasets generated by Integrated Health Interview Series (IHIS) available for public use. IHIS is a harmonized set of data and documentation based on material originally included in the public use files of NHIS and distributed for free over the Internet [15]. IHIS dataset provides easier data management while keeping all the original information of NHIS. NHIS is conducted periodically by the Centers for Disease Control and Prevention's (CDC) National Center for Health Statistics (NCHS). The core questionnaires provide information on demographics, health status, health behaviors, and health care access and utilization. CAM as part of the additional or supplemental information is collected on randomly selected one adult (18 years or older) and one child (0–17 years old) in each family on certain years in 2007. The 2007 CAM supplement included questions on 36 types of CAM therapies used in the United States. These therapies included 10 types of provider-based CAM therapies (e.g., acupuncture), as well as 26 other nonprovider-based CAM therapies (e.g., natural products, special diets, and movement therapies). Detailed description of this survey is seen in the National Health Statistic Report [3].

### 2.2. Measures Selected

For this study, a number of sociodemographic variables were selected and recoded for the purpose of statistical analysis. Those variables included, gender (male and female), age (18–40 years, 41–64 years, and 65 years and above), race (White, Black, Asian, and other), Hispanic origin (yes or no), place of birth (in USA or not in USA), US citizenship (yes or no), educational attainment (less than 12 grades, high school graduates, some college, college graduate, and above), marital status (currently married or not), region of residence (northeast, north central/midwest, south, and west), and self-rated health (poor to good or great to excellent).

Acupuncture use measures selected for our study included the following categories. (1) Use of acupuncture: respondents were asked if they had ever used acupuncture for their own health. Those who answered yes were referred as “ever acupuncture user.” Those respondents were then asked if they had used acupuncture in the previous 12 months. Those who responded affirmatively were referred as “recent user.” Those who responded negatively were referred as “former user.” (2) Acupuncture visit information: respondents were asked the number of visits for acupuncture treatment and the cost per visit. Note that a list of 81 conditions was provided in NHIS for respondents to choose for the condition they used acupuncture for. Those conditions were not selected for our analysis. (3) Responses to reason of using acupuncture in the past 12 months included the following: for specific health problems, for enhanced energy, for improved immune function, for general wellness, health care provider recommended, medical treatment is expensive, medication treatment did not help, and family and friends recommended. (4) Acupuncture use and other treatment: respondents were asked if they had other treatments including counseling, nonprescription medications, prescription medications, physical therapy, surgery, or no conventional treatment for conditions that were reported to be treated using acupuncture. If so, they were then asked the timing of having it (before, about the same time, or after having acupuncture). (5) Reasons of not using acupuncture: response to the reasons of never having used acupuncture include “*acupuncture is too expensive; not needed; not proven to work or not safe”* or “*acupuncture does not work*;* did not know about acupuncture or never thought about*; *health care provider said not to; No reason or some other reasons*.*”* Response to the reasons of not having used acupuncture in the past 12 months were similar to the ones in ever acupuncture use, except for replacing “*acupuncture is not safe*” with “*acupuncture has side effects*” and removing the question “*did not know about it*.”

### 2.3. Samples

In NHIS 2007, interviews were completed in 29,266 households, which yielded 75,764 persons in 29,915 families and a household response rate of 87.1% [3]. Our analysis was based on a sample from 23,393 completed adult interviews indicating an individual response rate of 67.8% [3]. Three sets of samples were used in our analysis. The first one included all nonusers (*N* = 21,861, i.e., 23,393 total respondents minus 1,532 acupuncture users) to assess the reasons of having never used acupuncture. The second one included the former acupuncture users (*n* = 1,188, i.e., 1,532 acupuncture user minus 344 recent users) to explore the reasons of not using acupuncture in the past 12 months. The third sample includes recent acupuncture users (*n* = 344) who provided valid responses related to their use of acupuncture such as reasons of visit and payment per visit.

### 2.4. Statistical Analysis

All analyses were performed using Stata 9.0 (Stata Statistical Software: Release 9. College Station, TX: StataCorp LP). All analyses used the IHIS individual-level sample weights [15]. Stata survey comments were used for the complex survey sample design. Overall analysis included examination of the weighted distribution of variables in the analytic sample and the weighted prevalence of recent acupuncture use by the sociodemographic variables. Patterns of use among recent users, such as number of visits and cost, were examined using weighted univariate and bivariate tabulations. Weighted logistic regression was performed on recent acupuncture use to determine which sociodemographic variables were significantly associated. For each analysis, a new analytic sample was created in order to exclude individuals with missing data on the variables involved. The significance level was set as 0.05.

## 3. Results

### 3.1. Acupuncture Use and Reasons

There was a 0.3% increase, representing a little over 1 million adults of recent acupuncture users from 2002 (1.1% of the population, i.e., 2.13 million American adults) to 2007 (1.4%, i.e., 3.14 million American adults) [7]. The ever acupuncture users increased from 4.2% of to 6.3% of the population, representing 8.19 million and 14.01 million in 2002 and 2007, respectively [7]. Of the recent users, 25% saw the acupuncturist only one time and 43.8% visited 2 to 5 times, 16.5% visited 6–10 times, and 14.6% visited more than 10 times. The average out of pocket cost per visit for recent users was $103.03 ± 12.50 (95%CI $78.44–$127.61) and the median cost was $48.3 (95%CI $38.39–$49.50). The top three reasons of recent visit to an acupuncturist were for a specific health problem (87.1%), medical treatment did not help (47.0%), and family and friends recommended (45.1%) or health care provider recommended (27.3%). Acupuncture also was used for general wellness (42.3%), enhanced energy (24.0%), and improved immune function (21.7%). About 10% of recent visits were because of medical treatment being expensive. Recent acupuncture users reported that a conventional medical professional (55.0%), particularly medical doctors (52.8%), were the resources from whom they were informed of acupuncture. Health care providers from other disciplines such as nurse practitioner (3.3%), doctor of osteopathy (3.1%), psychiatrist (1.5%), dentist (1.1%), and psychologist (0.9%) were not the main resources of acupuncture information. No recent acupuncture user reported being informed of acupuncture use from their pharmacist.

### 3.2. Sociodemographic Characteristics of Acupuncture Users

Table 1 presents the sociodemographic characteristics of acupuncture users including former and recent users. The average age of former, recent, and nonacupuncture user were 51.9 ± .64, 48.1 ± .95, and 45.43 ± .14 years old, respectively. The former users were statistically significantly older than the recent user and nonuser (*P* < .01). Of the recent users, more than half (65%) of them were females. A majority of them reported being non-Hispanic origin (89.9%) or White (82.2%). Nearly half (47%) had college or higher education level. Chi-square tests showed that age, sex, race, education level, and residence region were statistically related to recent acupuncture use when compared with former use. After adding nonusers in the comparison, all sociodemographic characteristic except for marital status became significant. Furthermore, when compared with nonusers, weighted multinomial logistic regression (Table 2) revealed that among the recent users, being middle age (OR = 1.76 for 41–64 age group), female (OR = 1.83), Asian (OR = 1.56), US citizen (OR = 1.34), having higher education level (OR = 2.05 for high school graduate, OR = 4.22 for some college, OR = 6.85 for college and above), having poorer self-rating of health (OR = 1.07), and living in West (OR = 1.55) were associated with recent acupuncture use. While among the former users, being middle age (OR = 2.21 for 41–64 age group) and old age (OR = 2.39 for age 65+), female (OR = 1.29), Asian (OR = 1.52), having higher education level (OR = 1.84 for high school graduate, OR = 2.93 for some college, OR = 3.86 for college and above), having poorer self-rating of health (OR = 1.05), and living in West (OR = 1.77) were associated with acupuncture use. The main difference of the characteristics of those two groups is that US citizens were more likely to be recent users while old age group were likely to be former users.

### 3.3. Acupuncture and Other Treatment

**Figure 1:**
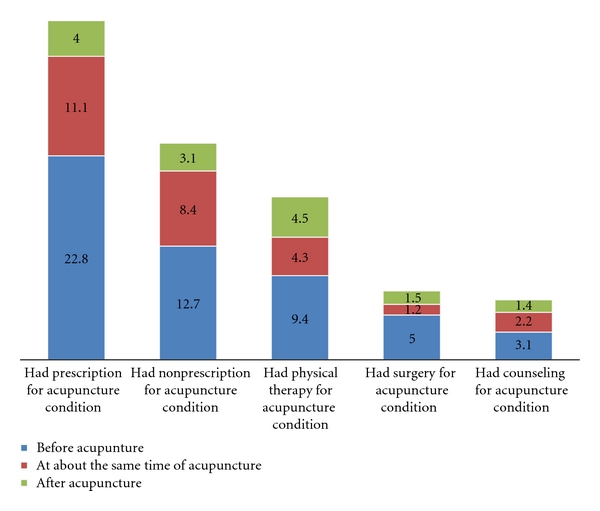
The percentage of recent acupuncture users' use of other treatment and the timing of its use (*n* = 344).

As shown in Figure 1, of the recent acupuncture users, about 38% of acupuncture users had prescription medicine, 24% had nonprescription medicine, 18% had physical therapy, 7.7% had surgery, and 6.7% had counseling for the acupuncture conditions. Consistently across all the other treatments that the patients used, a majority of them had the treatment before having the acupuncture treatment. In addition, 31% of the recent acupuncture users indicated that they had nonconventional treatment (format not specified in the survey) for the condition that the patient used acupuncture for.

### 3.4. Reasons for Not using Acupuncture

**Figure 2:**
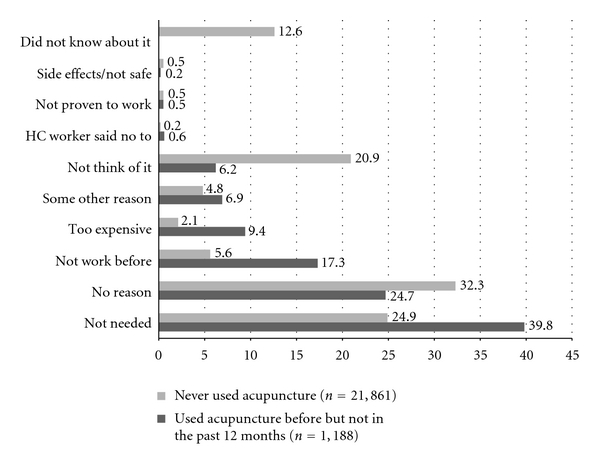
The percentage of the non- and former acupuncture users' reasons of not using acupuncture.

 Illustrated in Figure 2, the main reasons for not using acupuncture were “acupuncture is not needed” and “no particular reasons” for both recent (39.8% and 24.7%) and ever uses (24.9% and 32.3%). “Acupuncture did not work before” (17.3%) and “it is too expensive” (9.4%) were addressed by recent acupuncture users. “Never thought of acupuncture” (20.9%) or “did not know about it” (12.6%) were highlighted by those who never used acupuncture. Health care worker's negative opinion, evidence of acupuncture effectiveness, and its side effects or safety appeared to not have much impact on either ever or recent use (all <1%).

## 4. Discussion

### 4.1. Acupuncture Use and Reasons

 Our finding mainly reflects the increasing use of acupuncture among the American population, indicating more people now accept acupuncture treatment for their health care as compared to a few years ago. Acupuncture recent user increased from 0.4% of population in 1990 to 1.01% in 1998 [1], and from 1.1% in 2002 to 1.4% in 2007 [2, 3], representing 1% increase from 1990 to 2007. Based on this trend, we project a continuing but small (about 0.19 ~ 0.3%) steady increase of acupuncture users in NHIS 2012.

Our study showed that the majority of the patients used acupuncture for a specific condition, which is similar as the 2002 NHIS findings. Almost half of them sought this CAM modality because of conventional treatments not working. Given that the US health system is well equipped with advanced technology, adequate personnel, and established infrastructure, it is reasonable that acupuncture is considered helpful for a limited number of conditions nowadays [16]. We found that it is also common that people will choose acupuncture only after conventional treatments failed. Although acupuncture could serve as a valuable complementary treatment for some conditions that conventional treatments could not help, the best timing for acupuncture treatment may have passed when the patient finally visits an acupuncture practitioner after trying everything else. Such delay may decrease the effectiveness of acupuncture treatment. In addition, some patients are skeptical and may likely discontinue treatment if they do not feel results right away. It is particularly challenging for acupuncture practitioners in the USA to demonstrate effectiveness after one or two sessions of acupuncture treatment when often a series of acupuncture treatments are needed. This observation warrants future research on acupuncture treatment dosage, duration, and its effectiveness.

The new questions in NHIS 2007 revealed that some people used acupuncture to achieve general wellness, enhance energy, and improve immune function. This suggests that Americans are acknowledging acupuncture as a CAM modality that can help them in health promotion. Although acupuncture nowadays is used for a specific condition more frequently, it was developed to promote Qi (vital energy) flow and rebalances the system to achieve health or overall wellbeing according to Traditional Chinese Medicine (TCM) theory [6]. As the TCM theories are too abstract or philosophical for the general public to understand and there is no conclusive Western theory explaining acupuncture mechanisms, acupuncture practitioners may often find it very difficult to communicate with their American patients about how acupuncture works. We suggest that acupuncture practitioners use lay language to help the patients make informed decisions about their treatment plan by providing adequate treatment options in addition to acupuncture.

We cannot ignore the fact that nearly half of the respondents used acupuncture because their family or friends recommended it. Acupuncture practitioners may want to keep in mind that every single patient could be an advocate for acupuncture use. A treatment session could also be a good opportunity of providing appropriate acupuncture education. On one hand, for those who tried conventional treatment, acupuncture practitioners need to collect patient's conventional treatment history and discuss how acupuncture can help. On the other hand, for those patients who are frightened of the potential risks or adverse effects of conventional treatments, acupuncture practitioners need to encourage them to seek appropriate treatment if acupuncture cannot help them.

More than half the acupuncture patients were informed about this CAM modality by their conventional medical professionals, particularly medical doctors. This is encouraging as it may suggest that conventional health providers are becoming more familiar with acupuncture. This communication between conventional health providers and their patients is encouraged as we have seen a trend in implementing information about the safe use of CAM into the curriculum of conventional medical education [17].

### 4.2. Sociodemographic Characteristics of Acupuncture User

Compared with 2002 NHIS, 2007 NHIS data showed some changes regarding to sociodemographic characteristics of acupuncture recent user including more female users (2002, 52% versus 2007, 65%), more middle age and older users (40 yr+, 2002, 59.6% versus 2007, 69.7%), and fewer users in northeast and north central/midwest but more users in south and west. Despite these changes, the findings demonstrated that the acupuncture users' sociodemographic characteristics in 2007 were generally similar as that in 2002, that is, a typical acupuncture recent user would be an Asian female, living in the west, having poorer self-reported health status rating, and with a higher level of education. It is worth noting that sociodemographic characteristics such as age should not be viewed as the sole predictor of acupuncture use; instead, similar as factors related to other health services, patient's socioeconomic status and availability and accessibility of acupuncture practice all play key roles in acupuncture use as well. For instance, having more acupuncture users in the west may simply reflect the census fact that California had the largest Asian population [18]. Additionally, more acupuncture schools and acupuncturists located in the west, particularly in California, increase the exposure of such CAM modality, which probably contributes to the higher use rate in this region [19, 20]. Moreover, better health insurance policies in the west that cover CAM therapies, including acupuncture, such as the “Every Category of Provider Law” in Washington state may contribute to more acupuncture use. We expect some changes of the user profile when acupuncture becomes more available and accessible in other regions of USA.

### 4.3. Acupuncture and Other Treatment

Our findings showed that about two-thirds of acupuncture patients have used various conventional treatments such as prescription medication, surgery, or physical therapy. This indicates that more patients use conventional treatment combined with acupuncture treatment, or in other words, they use acupuncture treatment as part of integrative medicine. It might be a future direction of acupuncture practice in USA to be used in conjunction with other treatment modalities which may provide synergistic effects. Acupuncturists' adequate biomedicine training, which is required for national certification of acupuncture [21], may help facilitate their communication with the conventional health providers. Such communication may promote better collaboration and inclusion of acupuncture as a therapeutic modality and should be encouraged when possible. A previous study showed that although acupuncturists and western physicians both spend average 25 hours per week with patients, acupuncturists see fewer patients and spend double or triple the time with each one [22]. There is an opportunity that acupuncture practitioners may catch something missed by the physicians; therefore, greater communication between acupuncture practitioners and conventional health providers is needed to improve the quality of health care for their mutual patients. However, there are several barriers identified that make it difficult to carry out such communication successfully mostly due to differences in underlying principles and concepts of western medical practice and acupuncture [23, 24], or lack of familiarity or skills of using communication channels effectively [25]. Although educational initiatives that focus on improving knowledge, attitudes and communication skills may help [26], whether or not such initiatives can truly help overcome the communication gap is still questionable and further study is needed to assess their effectiveness and impact.

### 4.4. Reasons for Not using Acupuncture

A surprising finding was that safety, effectiveness, and healthcare providers' negative opinion appeared to not affect people's decision of whether or not to use acupuncture. Of the main reasons for not using acupuncture, unawareness or unfamiliarity ranked at the top. This suggests that acupuncture may not be as popular as it appears. Acupuncture was introduced into the USA in early 1970s [6]. Although Americans hear about it from different media sources, many are probably unclear about how acupuncture can help them. Education provided by trained acupuncture practitioners or health care providers is needed to help the general public better understand this modality. For instance, they need to know that acupuncture can help a variety of conditions and promote overall health, and also to be aware that acupuncture does not always work for every condition or disorder. Our findings showed that 17% of previous acupuncture users did not use this in the past 12 months because it “did not work before.” One explanation may be that acupuncture was not the appropriate treatment for his/her condition. Another possible explanation is that the patients did not give sufficient time for acupuncture to start to work.

In 2007 NHIS, some previous acupuncture users considered cost as one of the reasons of not using acupuncture anymore. As shown in our findings, the average out of pocket cost per acupuncture visit is $103 and a median cost is $48. This positive skewed distribution indicates that few patients paid much more than most of the users. Regardless, $48 out-of-pocket cost per acupuncture visit is usually more than the copay for a regular conventional doctor visit. In addition, when compared with other CAM modalities, median out-of-pocket cost per acupuncture visit ranked as number two following naturopathy (median cost $63) [8]. Those figures all indicate that acupuncture could be “too expensive” for some patients. As we mentioned earlier, if acupuncture can be covered by health insurance, then cost might not be a reason of not using acupuncture.

### 4.5. Limitations

Similarly as reported on acupuncture use among American adults in 2002 [7], one should interpret relevant findings from this NHIS based study with caution because of the cross-sectional nature of the data and relative small sample size. We cannot draw conclusions about possible causal pathways between two explored variables nor explore time trends. Moreover, the relatively small number of recent acupuncture users may result in unstable estimates of reasons for acupuncture use or mean cost of an acupuncture visit. It also limits further exploration on a subset of samples with particular characteristics. In addition, we only explored sociocharacteristics and reasons of the acupuncture use, while socioeconomic status should be taken into consideration in the future research to obtain a more holistic picture. Furthermore, patients' use or not use of acupuncture may also be related to a specific health condition which needs to be assessed in the future research as well.

## 5. Conclusions

Despite those limitations, our findings demonstrate that people not only use acupuncture as a complementary and alternative approach to conventional treatment, but also use it as a preventive modality to promote general health. That may explain why acupuncture has become more popular in the USA during the past decade. However, the low percentage of overall acupuncture use and slow growth trend (from 0.4% in 1990 to 1.4% in 2007 among American population) indicates that the acupuncture market may not be saturated yet. Lower cost, insurance coverage, and raising awareness of acupuncture may attract more acupuncture users. Although effectiveness and safety appeared not to affect patients' decision making for acupuncture use, healthcare providers, including acupuncture practitioners, should be equipped with such knowledge to better educate their patients. We expect to continue to see more patients turn to acupuncture treatment in the future and urge acupuncture practitioners to be better prepared to communicate with their patients and their conventional medicine colleagues in order to provide complete and safe holistic care to benefit patients.

## Figures and Tables

**Table 1:** Socio-demographics of the acupuncture users and non-users.

Socio-Demographics	Former User(*n* = 1,188)	Recent user(*n* = 344)	Non-User(*n* = 21,861)
Age^∗∗††^ (Mean ±linearized standard error)	51.9 ± .64	48.14 ± .95	45.43 ± .14
18–40	23.9	30.3	43.1
41–64	54.5	58.1	40.9
65+	21.6	11.6	16.9
Sex^∗††^			
Female	57.3	65.0	51.2
Male	42.7	35.0	48.8
Hispanic Origin^††^			
Non-Hispanic Origin	89.6	89.9	86.4
Hispanic Origin	10.4	10.1	13.6
Race			
White	81.8	82.2	80.9
Black	5.5	5.0	12.2
Asian	9.3	11.2	4.3
Other	3.4	1.5	2.5
Citizenship^†^			
US citizen	93.0	92.8	91.1
Not US citizen	7.0	7.2	8.9
Birth Place^†^			
Born in US	81.3	80.4	83.9
Not Born in US	18.7	19.6	16.1
Highest Education**			
<= Grade 12	6.9	4.4	14.8
High School graduate	23.9	17.5	31.4
Some college	30.8	31.1	28.0
College and above	38.4	47.0	25.8
Health Status^††^			
Very good-excellent	51.1	52.5	61.3
Poor-good	48.9	47.5	38.7
Region**			
Northeast	16.0	19.5	17.1
North Central/Midwest	20.6	15.5	24.4
South	27.5	27.8	37.3
West	35.9	37.2	21.2
Marital Status			
Currently Married	58.2	56.6	55.6
Not married	41.8	43.4	44.4

Former and recent users: *P < .05, **P < .01.

Former, recent and non-users: ^†^ *P* < .05, ^††^ *P* < .01.

**Table 2:** Weighted multinomial logistic regression of sociodemographic characteristics of recent and former acupuncture user versus nonusers.

Sociodemographics (*N* = 23,393)	Relative Risk Ratios (95% CI)	Relative Risk Ratios (95% CI)
	Recent users	Former users
Age (18–40)		
41–64	1.76 (1.29–2.41)***	2.21 (1.79–2.74)***
65+	0.96 (.61–1.52)	2.39 (1.87–3.05)***
Female	1.83 (1.39–2.41)***	1.29 (1.10–1.52)**
Hispanic origin	.90 (.57–1.42)	.90 (.69–1.17)
Race (White)		
Black	.45 (.25–.82)*	.53 (.41–.67)***
Asian	1.56 (1.00–2.45)	1.52 (1.13–2.03)**
Other	.60 (.26–1.40)	1.42 (.91–2.24)
US citizen	1.34 (1.09–1.65)**	1.04 (.89–1.23)
US born	0.80 (.56–1.16)	.83 (.65–1.04)
Education (<12 years)		
HS graduate	2.05 (1.06–3.95)*	1.84 (1.38–2.48)***
Some college	4.22 (2.23–8.01)***	2.93 (2.19–3.91)***
College above	6.85 (3.65–12.88)***	3.86 (2.91–5.11)***
Health status poor to good	1.07 (1.03–1.09)***	1.05 (1.03–1.07)***
Not married	1.00 (.99–1.02)	1.00 (.99–1.01)
Region (Northeast)		
North central/midwest	.59 (.38–.93)*	.95 (.73–1.23)
South	.76 (.52–1.11)	.88 (.70–1.11)
West	1.55 (1.08–2.23)*	1.77 (4.43–2.19)***

*P < .05, **P < .01, ***P < .001.
